# HIV/AIDS Mortality in the Eastern Cape Province of South Africa, 2000–2023: Trends, Age–Sex Composition, and a Persistent Provincial Burden

**DOI:** 10.3390/tropicalmed11070205

**Published:** 2026-07-22

**Authors:** Tronic Sithole, Ziphelele Peter, Ayanda Princess Myeni

**Affiliations:** 1 School of Public Health, Faculty of Medicine and Health Sciences, Walter Sisulu University, Mthatha 5100, South Africa; zpeter@wsu.ac.za; 2 Global Centre for Human Resources for Health Intelligence, Walter Sisulu University, East London 5247, South Africa; 3 Society and Health Research Institute, Faculty of Medicine and Health Sciences, Walter Sisulu University, Mthatha 5117, South Africa; 4 School of Social Sciences, University of KwaZulu-Natal, Durban 4000, South Africa; ayandamyeni136@gmail.com

**Keywords:** HIV/AIDS mortality, eastern cape, age–sex disaggregation, AIDS epidemic, South Africa

## Abstract

The Eastern Cape of South Africa carries the highest crude AIDS mortality rate nationally (135.86 per 100,000 in 2023), yet a province-specific, age-sex disaggregated analysis extending beyond 2012 does not exist. This study examined AIDS mortality trends in the Eastern Cape from 2000 to 2023. A quantitative ecological design was employed using secondary analysis of modelled estimates from the Thembisa Provincial HIV Model version 4.8. Annual AIDS deaths across nine age–sex subgroups and crude AIDS mortality rate were extracted from the Eastern Cape, 2000–2023, with provincial comparisons for 2023. Total AIDS deaths declined by 72.1% from 33,301 in 2005 to 9300 in 2023. Child deaths fell 96.1% from peak. Male youth deaths (15–24) plateaued after 2015. Adults aged 50 and older constituted 24.1% of deaths in 2023, with numbers increasing since 2019. Despite substantial gains, three unresolved gaps persist: a male youth mortality plateau, an ageing epidemic burden, and the highest provincial AIDS mortality rate nationally. Targeted differentiated interventions are urgently needed.

## 1. Introduction

South Africa continues to carry one of the world’s largest HIV/AIDS epidemics, accounting for a substantial share of people living with HIV in sub-Saharan Africa [1]. Over the past two decades, antiretroviral therapy (ART) rollout from 2004, universal test-and-treat policies, and combination prevention strategies have contributed to sustained reductions in AIDS-related mortality at the national level [2]. Pillay-van Wyk et al. [3] provided the most comprehensive empirical analysis of these national trends, reporting that HIV/AIDS deaths peaked at 283,564 in 2006 and had almost halved by 2012, with older men identified as a subgroup of particular concern. However, their analysis concluded in 2012, and province-specific, age–sex disaggregated characterisations of AIDS mortality trajectories extending to the current decade have not been published.

The Eastern Cape presents a specific and underexamined case within this national epidemic. The province is predominantly rural, characterised by high rates of poverty and unemployment, persistent deficits in health infrastructure, and poor geographic access to services, particularly in the former homeland districts of OR Tambo and Alfred Nzo [4]. Provincial ART coverage has improved substantially, from 67.8% in 2017 to 83.5% in 2022 [5], yet this masks marked district-level variation: ART use in 2022 ranged from 69.4% in Nelson Mandela Bay to 92.0% in Alfred Nzo, and viral load suppression among men aged 15–49 (65.4%) remained well below that among women in the same age group (83.9%) [6]. These structural conditions reduce ART programme effectiveness in ways not captured by national-level analyses. Young men who avoid health facility contact, and older adults ageing into their sixth decade with complex comorbidities from chronic HIV infection and long-term ART exposure [6], have neither been characterised for the Eastern Cape nor documented in the published literature. Recent UNAIDS reporting illustrates the scale of the province’s ongoing paediatric burden alone: an estimated 24,856 children aged 0–14 years were living with HIV in the Eastern Cape in 2023, with 1360 newly infected and 255 AIDS-related child deaths in the same year, prompting the launch of the province’s first Global Alliance chapter to accelerate paediatric HIV elimination efforts in late 2024 [7].

A further gap exists in the comparative provincial literature. Pillay-van Wyk et al. [3] documented provincial variation in HIV/AIDS mortality rates to 2012, but whether the Eastern Cape has since emerged as the province with the highest crude AIDS mortality rate despite not having the largest epidemic has not been established. Such a finding would implicate health system performance gaps rather than epidemic size as the driver of excess mortality.

This study uses modelled outputs from the Thembisa Provincial HIV Model v4.8 to address these gaps. The objectives are to: (1) describe temporal trends in total AIDS deaths and crude AIDS mortality rate in the Eastern Cape, 2000–2023; (2) characterise the shifting age–sex composition of AIDS deaths; (3) identify specific subgroup mortality gaps; and (4) benchmark the Eastern Cape against all other provinces in 2023.

## 2. Materials and Methods

### 2.1. Study Design

This study employed a quantitative ecological design using secondary analysis of South Africa’s nationally validated, province-level modelled HIV/AIDS mortality estimates. The analysis examined temporal trends in AIDS deaths across age–sex subgroups in the Eastern Cape between 2000 and 2023 and compared provincial mortality indicators in 2023.

### 2.2. Data Source

HIV/AIDS mortality estimates were obtained from the Thembisa Provincial HIV Model version 4.8 [8], developed by the Centre for Infectious Disease Epidemiology and Research (CIDER), University of Cape Town. Thembisa is a deterministic, compartmental model of HIV transmission, disease progression, and mortality that generates province-specific annual estimates for all nine South African provinces and the national aggregate from 1985 to 2030. The model integrates data from multiple sources, including antenatal clinic surveillance, population-based surveys, demographic data, and national HIV programme statistics from the District Health Information System (DHIS). Version 4.8 incorporates updated calibration inputs from the 2021 and 2022 population-based HIV impact assessments [2,9], and the provincial estimates used in this study are calibrated directly against province-level antenatal sentinel and household survey data for the Eastern Cape rather than derived by disaggregating a single national calibration; the model is therefore not simply assuming national generalisability to the province. The model has been widely used in peer-reviewed research and national HIV surveillance [3,10].

### 2.3. Study Population

The study population was the resident population of the Eastern Cape across all age groups, stratified by sex, for the period 2000–2023. The primary outcome population for age-sex disaggregated analysis consisted of eight subgroups: boys and girls aged 0–14 years; males and females aged 15–24 years; males and females aged 25–49 years; and men and women aged 50 years and older. For the provincial benchmarking analysis, the study population comprised the resident populations of all nine South African provinces and the national aggregate.

### 2.4. Study Variables

The primary outcome was annual AIDS deaths in the Eastern Cape, expressed as absolute counts disaggregated by age–sex subgroup for each year from 2000 to 2023. Additional indicators included total AIDS deaths, crude AIDS mortality rate per 100,000 population, and AIDS deaths in adults aged 15 years and older by sex. For comparative analysis, crude AIDS mortality rates and additional mortality indicators were extracted for all nine provinces and the national aggregate for 2023. The proportional share of each age–sex subgroup in total annual AIDS deaths was calculated for 2000, 2005, 2010, 2015, and 2023.

### 2.5. Data Analysis

Descriptive statistical analysis was conducted to characterise trends over the full study period. The percentage change from the epidemic peak (2005) was calculated for each age–sex subgroup. Provincial comparisons were cross-sectional, using 2023 estimates ranked by crude AIDS mortality rate. Data were extracted from the Thembisa provincial output workbook (ProvOutput4_8_final2.xlsx) using Python v3.11 with the openpyxl library. All figures were produced using matplotlib v3.9. The workbook provides mean values together with 95% uncertainty intervals (lower and upper bounds) for each indicator; the mean values for the nine age–sex subgroups (boys and girls 0–14; males and females 15–24, 25–49, and 50 and older) and total AIDS deaths were extracted directly as reported, with no additional transformation, smoothing, or standardisation applied beyond the percentage-change and proportional-share calculations described above. Crude AIDS mortality rates were taken as reported in the workbook rather than recalculated from raw death and population counts. Age–sex subgroup trends (Results 3.1–3.4) are reported as means only, consistent with the study’s descriptive design; 95% uncertainty intervals were additionally extracted for the crude AIDS mortality rate to support the provincial benchmarking comparison (Table 1).

### 2.6. Justification for Study Period

The period 2000–2023 was selected to capture the full arc of the Eastern Cape’s AIDS epidemic from the pre-peak growth phase through the ART-driven decline and the most recent period for which validated Thembisa v4.8 estimates are available. The year 2000 provides a meaningful pre-ART baseline; ART rollout began nationally in 2004 [3]. The year 2023 represents the most recent year for which retrospective modelled outputs are available from Thembisa v4.8.

### 2.7. Ethical Considerations

The data used in this study are publicly available, aggregated, and modelled epidemiological estimates derived from the Thembisa Provincial HIV Model v4.8. The dataset contains no identifiable individual-level information. Ethical review and approval were waived for this study as the analysis was based exclusively on anonymised, population-level modelled outputs constituting a secondary analysis of publicly available national estimates.

## 3. Results

### 3.1. Overall AIDS Mortality, 2000–2023

**Figure 1:**
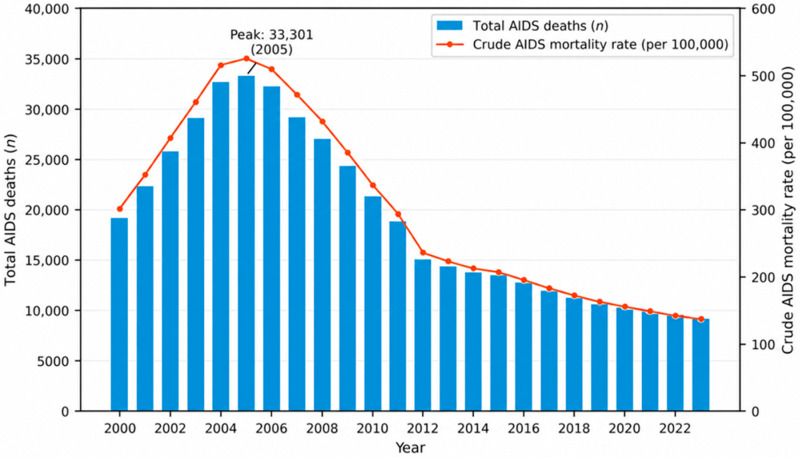
Total AIDS deaths (bars, left axis) and crude AIDS mortality rate per 100,000 population (line, right axis), Eastern Cape Province, 2000–2023. Source: Johnson [8].

Total AIDS deaths in the Eastern Cape peaked at approximately 33,301 in 2005, when the crude AIDS mortality rate reached 525.18 per 100,000 population (Figure 1). The national rollout of ART from 2004 drove a sustained decline: deaths fell to 21,406 in 2010, 13,593 in 2015, and 9300 in 2023, representing a 72.1% reduction from the epidemic peak. The crude AIDS mortality rate declined correspondingly to 135.86 per 100,000 by 2023. The rate of decline has decelerated sharply in the most recent period, falling by an average of 25.7 per 100,000 per year between 2005 and 2010, compared with only 5.4 per 100,000 per year between 2019 and 2023. This deceleration suggests a slowing pace of decline in the most recent period; possible explanations are considered in the Discussion.

### 3.2. Age–Sex Composition: A Structural Transformation

**Figure 2:**
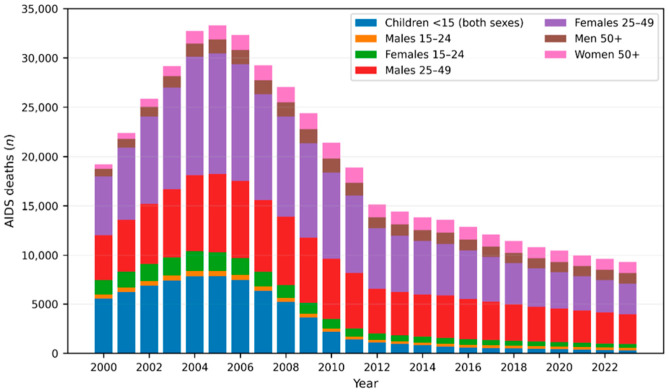
Age–sex disaggregated AIDS deaths, Eastern Cape Province, 2000–2023. Source: Johnson [8].

Figure 2 shows the shifting age–sex structure of AIDS deaths across the study period. In 2000, the burden was shared principally among women aged 25–49 years (31.2% of all AIDS deaths), men aged 25–49 years (24.0%), and children under 15 years (combined 28.9%). By the epidemic peak in 2005, women aged 25–49 dominated, accounting for 36.7% of all AIDS deaths. This feminisation of peak mortality is consistent with national patterns of elevated HIV acquisition risk among women of reproductive age documented elsewhere [11]; possible contributing mechanisms are considered in the Discussion. As ART coverage expanded, the child share declined from 28.9% in 2000 to 3.3% in 2023, the single most dramatic shift across all subgroups. By 2023, adults aged 50 and older represented a rising 24.1% of all AIDS deaths.

### 3.3. The Male Youth Mortality Plateau

**Figure 3:**
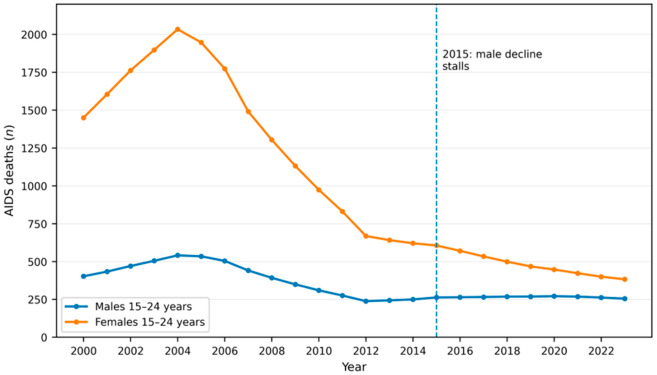
AIDS deaths in young adults aged 15–24 years by sex, Eastern Cape Province, 2000–2023. The dashed vertical line marks 2015, after which male deaths plateau while female deaths continue to decline. Source: Johnson [8].

Figure 3 identifies a critical and unresolved gap in the Eastern Cape’s HIV response. AIDS deaths in males aged 15–24 years declined from 534 in 2005 to 262 in 2015, tracking the overall ART-driven trend. From 2015 onward, this decline stalled: deaths in this group were 268 in 2019, 271 in 2020, and 255 in 2023, effectively unchanged over eight years. This plateau is absent in every other age–sex group. The female counterpart group continued to decline from 606 in 2015 to 382 in 2023, a group that had previously recorded a substantially higher mortality rate than young men and has now converged towards near-parity with the male plateau, a shift that itself merits dedicated investigation into the prevention and treatment gains achieved among young women in this period. This divergence extends the concern raised by Pillay-van Wyk et al. [3] regarding male mortality stagnation and suggests that the problem has now reached younger male cohorts in the Eastern Cape; possible drivers are considered in the Discussion.

### 3.4. The Ageing Epidemic

**Figure 4:**
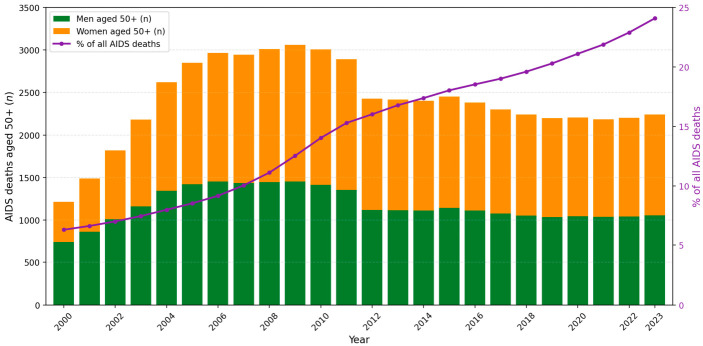
AIDS deaths in adults aged 50 years and older by sex (bars, men and women, left axis) and their combined proportional share of all AIDS deaths (line, right axis), Eastern Cape Province, 2000–2023. Source: Johnson [8].

Figure 4 documents a second unresolved gap: adults aged 50 and older are the only subgroup in the Eastern Cape in which AIDS deaths are increasing as a proportional share of all AIDS deaths, with a modest absolute increase. Deaths in men aged 50 and older rose from approximately 1037 in 2019 to 1057 in 2023, and in women from approximately 1162 to 1183, modest absolute increases against a backdrop of overall declining AIDS mortality. Their combined share of all AIDS deaths grew from 6.3% in 2000 to 24.1% in 2023—the more reliable indicator of this shift given the small absolute numbers involved. This growing 50-and-older burden is itself a marker of ART programme success: it reflects people living with HIV surviving into older age rather than dying prematurely, and men and women who acquired HIV in the epidemic’s peak years now ageing into their sixth decade. This was not visible in the 2012 endpoint of Pillay-van Wyk et al. [3] and has not previously been documented for the Eastern Cape. Deaths in this age group are close to evenly split between men and women (1057 versus 1183 in 2023) despite women constituting a substantially larger share of people living with HIV nationally; regional data indicate that treatment coverage and viral suppression among adult men continue to lag behind those among adult women [12], and the near-parity in absolute deaths observed here is consistent with older men being disproportionately under-treated relative to their share of the epidemic. The mechanisms and service-delivery implications of this shift are considered in the Discussion.

### 3.5. The Eastern Cape’s Comparative Mortality Disadvantage

Table 1 benchmarks crude AIDS mortality rates and related indicators across all nine provinces and the national aggregate in 2023. The Eastern Cape’s rate of 135.86 per 100,000 (95% CI 118.74–152.87) was the highest of all provinces, exceeding Mpumalanga (128.23, 95% CI 114.45–142.86), KwaZulu-Natal (126.92, 95% CI 114.40–141.42), and the national average (85.64). The 95% confidence intervals for the Eastern Cape, Mpumalanga, and KwaZulu-Natal overlap substantially, so this ranking should not be read as statistically distinguishing the Eastern Cape from these two provinces specifically. The Western Cape recorded the lowest rate at 29.45 per 100,000 (95% CI 26.57–34.44), a range that does not overlap with the Eastern Cape’s. KwaZulu-Natal, which carries a substantially larger epidemic, achieves a markedly lower crude AIDS mortality rate. This pattern is consistent with health system performance, rather than epidemic size, as a contributor to the Eastern Cape’s excess mortality. However, the ecological design of this study cannot establish this causally [4].

## 4. Discussion

This study estimated that AIDS deaths in the Eastern Cape declined by 72.1% from a peak of approximately 33,301 in 2005 to 9300 in 2023. This trajectory is broadly consistent with the national pattern documented by Pillay-van Wyk et al. [3], who reported a national peak of 283,564 deaths in 2006 and a halving of this figure by 2012. The current analysis extends this evidence eleven years further, identifies three specific and unresolved mortality gaps, and demonstrates that the Eastern Cape now carries the highest crude AIDS mortality rate of any province in South Africa. The marked deceleration in the pace of decline after 2015 is consistent with the most accessible gains from ART expansion having already been realised, with residual mortality now concentrated in population groups not yet fully reached by existing programmes, a pattern explored further in the subgroup findings below. The feminisation of peak mortality observed in 2005, when women aged 25–49 accounted for 36.7% of all AIDS deaths, is consistent with the higher per-act male-to-female transmission probability, earlier average age at sexual debut among women, and structural gender inequalities in HIV risk that have been documented nationally [11]; disaggregating the specific contribution of each of these mechanisms in the Eastern Cape context was beyond the scope of this ecological analysis.

South Africa’s most significant success against AIDS mortality was among children under 15. In the Eastern Cape, child AIDS deaths declined by 96.1% from the 2005 peak, consistent with Pillay-van Wyk et al. [3] who found this represented the nation’s greatest gain. This reduction is attributable to prevention of mother-to-child transmission (PMTCT) programme expansion and paediatric ART [13,14]. The residual 304 child AIDS deaths estimated for 2023 represent preventable losses in a province where PMTCT coverage remains below national benchmarks [15,16].

The plateau in AIDS deaths among males aged 15–24 since 2015 extends the concern raised by Pillay-van Wyk et al. [3] and Haal et al. [17] regarding male mortality stagnation, demonstrating that it now affects younger cohorts and has not improved over eight years of continued ART expansion. Cornell et al. [18] and Kanters et al. [19] attributed male mortality disadvantage to later ART initiation and more advanced disease at enrolment. In the Eastern Cape, these clinical patterns are compounded by geographic barriers, facility hours that conflict with employment, and masculinity norms discouraging health-seeking [4]. Community-based HIV testing, workplace-linked ART initiation, and peer-navigated adherence support must be implemented at scale in districts with the highest young male burden [20,21,22]. District-level survey data point to Nelson Mandela Bay and Buffalo City, the province’s two metropolitan districts, as priority sites: these recorded the lowest ART use of the six priority districts (69.4% and 79.4% respectively) despite their relatively better geographic access to services, suggesting that urban-specific barriers such as facility hours conflicting with employment may be more relevant here than the rurality-focused explanations that dominate the wider Eastern Cape literature; rural districts such as Alfred Nzo, however, recorded the highest ART use (92.0%) despite greater distances to facilities [6].

The ageing of the AIDS epidemic, with adults aged 50 and older now accounting for 24.1% of all AIDS deaths and their absolute numbers rising modestly, was not visible in 2012 and has not previously been documented at the provincial level [3,23,24,25]. Eke et al. [26] and Nakanjako et al. [27] have established that ageing with HIV in sub-Saharan Africa is associated with elevated multi-morbidity driven by chronic immune activation and cumulative ART toxicity. The Eastern Cape’s public health infrastructure is not configured to manage the complex polypharmacy-dependent care needs of older patients [8]. Integrated chronic disease management pathways incorporating HIV care for older adults are a present-tense planning priority.

The provincial benchmarking confirms that the Eastern Cape’s mortality burden is not simply a reflection of epidemic size. KwaZulu-Natal achieves a lower crude AIDS mortality rate in 2023 despite a larger absolute HIV burden. This finding replicates the provincial variation documented by Pillay-van Wyk et al. [28] and Achoki et al. [29] but reveals a shift in provincial ranking since 2012. The Eastern Cape’s comparative disadvantage is hypothesised to reflect health worker vacancies, supply chain failures, and poor geographic access as contributors to preventable deaths; this ecological analysis cannot confirm these mechanisms and testing them directly is a priority for future research [4].

This study has limitations. The Thembisa model generates modelled estimates subject to parameter uncertainty. Subsequently, 95% uncertainty intervals for the crude AIDS mortality rate were obtained from the provincial output workbook (Table 1): the Eastern Cape’s rate, while the highest point estimate nationally, is not statistically distinguishable from Mpumalanga or KwaZulu-Natal, though it remains clearly distinguishable from lower-burden provinces. The model does not disaggregate below the provincial level, precluding examination of sub-provincial heterogeneity between metropolitan municipalities and former homeland districts within the Eastern Cape. The ecological design precludes inference about individual-level risk factors. The analysis covers AIDS deaths exclusively and does not examine non-AIDS causes of death, including COVID-19-related excess mortality in 2020 and 2021. Disruption to HIV testing, ART initiation, and adherence support during the 2020–2021 COVID-19 pandemic period has been documented across South Africa. Any resulting interruptions in treatment in the Eastern Cape could plausibly have contributed to the deceleration in mortality decline and the persistence of the male youth plateau observed in this period. The modelled estimates used here cannot separate pandemic-related disruption from other structural drivers, and this remains an important area for further study.

## 5. Conclusions

HIV/AIDSmortality in the Eastern Cape has declined substantially since the ART rollout, most dramatically among children under 15. Despite this progress, the province enters 2023 with the highest crude AIDS mortality rate nationally, AIDS deaths among males aged 15–24 years unchanged since 2015, and AIDS deaths among adults aged 50 and older increasing. National-level epidemic gains documented since 2012 have not translated into comparable provincial improvements. Treatment and prevention programmes should, based on this evidence, prioritise reaching young men outside of conventional clinic-based pathways and develop age-sensitive, integrated chronic disease management for older adults ageing with HIV. These findings carry direct implications for the Eastern Cape provincial health authority’s strategic planning and for South Africa’s National Strategic Plan on HIV.

## Figures and Tables

**Table 1:** AIDS mortality indicators by province, South Africa, 2023. Eastern Cape values are in bold. Crude rate = AIDS deaths per 100,000 population. Deaths aged 50+ (%) = proportion of total provincial AIDS deaths in adults aged 50 years and older. Source: Johnson [8].

Province	Total AIDS Deaths (*n*)	Crude Rate/100,000	AIDS Deaths Males 15+ (*n*)	AIDS Deaths Females 15+ (*n*)	AIDS Deaths Children < 15 (*n*)	Deaths Aged 50+ (%)
South Africa (national)	52,964	85.64	27,078	24,197	1689	17.2%
**Eastern Cape**	**9300**	**135.86**	**4333**	**4663**	**304**	**24.1%**
Free State	2366	80.20	1192	1095	79	26.5%
Gauteng	10,104	62.61	5311	4195	598	27.3%
KwaZulu-Natal	15,150	126.92	7644	6704	802	23.5%
Limpopo	6256	95.05	2757	3202	297	27.0%
Mpumalanga	6328	128.23	3226	2925	176	22.8%
Northern Cape	1157	92.73	586	541	30	23.0%
North West	4794	121.39	2615	2006	173	27.5%
Western Cape	2160	29.45	1002	1052	106	20.3%

Source: Thembisa Provincial HIV Model v4.8 [8]. Bold row indicates Eastern Cape. Population denominators and 95% uncertainty intervals underlying these rates are provided in Table 1.

## Data Availability

The datasets analysed during the current study are publicly available from the Thembisa model outputs page hosted by the Centre for Infectious Disease Epidemiology and Research, University of Cape Town, at https://www.thembisa.org (accessed on 1 April 2025). The estimates used in this study are available in the public domain and can be accessed without restriction.
